# Determination of optimal load in the Wingate Anaerobic Test is not depend on number of sprints included in mathematical models

**DOI:** 10.3389/fphys.2023.1146076

**Published:** 2023-05-30

**Authors:** Kamil Michalik, Marcin Smolarek, Bartosz Ochmann, Marek Zatoń

**Affiliations:** ^1^ Department of Human Motor Skills, Faculty of Physical Education and Sport, Wroclaw University of Health and Sport Sciences, Wrocław, Poland; ^2^ Department of Physiology and Biochemistry, Faculty of Physical Education and Sport, Wroclaw University of Health and Sport Sciences, Wrocław, Poland

**Keywords:** wingate test, peak power output, force-velocity, anaerobic performance, braking force, all-out exercise

## Abstract

Determining the optimal load (OPT_LOAD_) in measuring mechanical peak power output (PPO) is important in assessment of anaerobic fitness. The main goals of this study were: 1) to examine estimated optimal load and PPO based on a force-velocity test and 2) to compare the PPO from the previous method with the Wingate Anaerobic Test (WAnT). The study involved 15 academic male athletes, aged 22.4 ± 2.3 (years), height 178.9 ± 6.8 (cm), and body weight 77.9 ± 12.2 (kg). They performed the 30-s WAnT (7.5% of body weight) during the first visit to the laboratory. Second to fourth session included a force-velocity test (FVT) involving three, 10-s all-out sprints. A randomized load ranging from 3 to 11 kg was used in each session for FVT. The OPT_LOAD_ and PPO were computed using quadratic relationships based on power-velocity (P-v) and power-percent of body weight (P-%BM) and including three, four, five and nine sprints from FVT. The results showed non-difference in OPT_LOAD_ [13.8 ± 3.2 (%BM); 14.1 ± 3.5 (%BM); 13.5 ± 2.8 (%BM); 13.4 ± 2.6 (%BM)] executed at three, four, five, and nine sprints (F_3,56_ = 0.174, *p* = 0.91, η^2^ = 0.01). The two-way ANOVA revealed that PPO were similar between tested models (P-%BM vs. P-v) independently from the numbers of sprints (F_3,112_ = 0.08, *p* = 0.99, η^2^ = 0.000). Moreover, the PPO measured in the WAnT (870.6 ± 179.1 W) was significantly lower compared with in P-v model (1,102.9 ± 242.5–1,134.2 ± 285.4 W) (F_4,70_ = 3.044, *p* = 0.02, η^2^ = 0.148). In addition, the PPO derived from P-%BM model (1,105.2 ± 245.5–1,138.7 ± 285.3 W) was significantly higher compared with the WAnT (F_4,70_ = 2.976, *p* = 0.02, η^2^ = 0.145). The findings suggest the potential utility of FVT for assessment of anaerobic capacity.

## Introduction

A sprint-based cycle ergometer test is a common method to assess anaerobic capacity (Praagh, 2007; Driss and Vandewalle, 2013). The 30-s, all-out Wingate Anaerobic Test (WAnT) is a frequently used cycle ergometer test, which uses an individual load in relation to the subject’s body weight (originally 7.5%) (Bar-Or, 1987; Castañeda-Babarro, 2021). The indices derived from this test include absolute and relative peak power output (PPO) [per kilogram of total body mass (BM) or lean body mass (LBM)], time to reach peak power output (tPPO), total performed work in the test (Wtot) and fatigue index (FI). These parameters are crucial in determining the physiological potential of athletes in many sports disciplines (Zupan et al., 2009; Alemdaroglu, 2012; Coppin et al., 2012; Chiarlitti et al., 2018; Nikolaidis et al., 2018) and effects of training intervention (Polczyk and Zatoń, 2015; Olek et al., 2018). Previous studies attempted to develop normative data tables for the WAnT, but used different load (Zupan et al., 2009; Coppin et al., 2012; Nikolaidis et al., 2018). Finally, various normative data from WAnT are available, mainly due to differences in loading and methodology, making it burdesome for asessment of data.

Researchers explored modification of time duration in WAnT to determine power output (Zając et al., 1999; Boraczyński et al., 2020; Hernandez-Belmonte et al., 2020). For example, Zając et al. (1999) compared 10- and 30-s bouts in assessing maximal anaerobic performance. They showed that PPO was higher and time to peak was shorter in the 10-s test, probably due to psychological factors. They suggested that shorter tests should be used in the PPO assessment, especially among strength-speed athletes, because their efforts are based on maximal-intensity activities dominated by the phosphagen ATP resynthesis system (Zając et al., 1999). Boraczyński et al. (2020) employed the 10-s WAnT at 7.5% body mass (BM) to determine the relationship of anaerobic capacity with measures of physiological capacities among professional soccer players. Hernandez-Belmonte et al. (2020) established the validity and sensitivity of two time durations (15-s and 20-s) in WAnT. Thus, the aforementioned studies suggested the utility of shorter duration in WAnT.

The inhibitory load in WAnT also plays a crucial role for accuracy in power output (Driss and Vandewalle, 2013). In the past, many studies focused on identifying the optimal load, expressed as a percentage of body weight (%BM) (Evans and Quinney, 1981; Dotan and Bar-Or, 1983; Patton et al., 1985; Vandewalle et al., 1987; Duche et al., 2002; Jaafar et al., 2014) or a percentage of lean body mass (%LBM) (Üçok et al., 2005). The traditional WAnT used the 7.5% BM load in children (Bar-Or, 1978). Subsequently, the 8.6% BM (Dotan and Bar-Or, 1983) and even 9.5% BM (Patton et al., 1985; Vandewalle et al., 1987) were proposed for adult men. In turn, Bar-Or (1987) recommended a load of 9% for non-training individuals and 10% for athletes. It is important to note that a 20% underestimaton in optimal load corresponds to a 5% disparity in actual maximal power (Driss and Vandewalle, 2013). Interesting results were provided by Jaafar et al. (2014), who compared effects of 8.7% and 11% of body weight and examined the reliability of these protocols. They reported higher peak and mean power production in the test with higher load and suggested that the load should be selected individually, especially in powerful athletes exceeding 15% of BM (Jaafar et al., 2014). In turn, Üçok et al. (2005) used the %LBM approach and verified the loads between 7.5% and 11% LBM. They showed that the optimal load for young untrained men is equal 10%-11% LBM, but did not determine precisely the optimal load expressed by %LBM, but only approximated. According to our best knowledge any previous research did not establish optimal load in the WAnT in regards to %LBM in academic athletes. Thus, it could add some new recommendations for maximal power measurement.

The force-velocity relationship test (FVT) performed on a stationary cycle ergometer consist of multiple all-out sprints for a short period 4–15 s (MacIntosh et al., 2003; Vargas et al., 2015; Jaafar et al., 2016; Nikolaidis and Knechtle, 2021) against different braking loads (Driss and Vandewalle, 2013). In the previous research two different approaches were applied. Firstly, loads are set to percentage of body weight (Vargas et al., 2015; Krüger et al., 2020). Secondly, based on absolute loads expressed in kilograms (Jaafar et al., 2016; Nikolaidis and Knechtle, 2021). The advantages of using this test include 1) the assessment of the ability to produce neuromuscular force by a relatively readily available, safe and reliable method; 2) the fact that working on a cycle ergometer is considered a well-known task that anyone can perform (Driss and Vandewalle, 2013; Rudsits et al., 2018). FVT allows to determine the linear relationship between braking force/torque and pedaling speed (cadence) and the multinomial relationship between power and speed/cadence (Driss and Vandewalle, 2013). The peak of the P-v curve refers to the peak power output (Samozino et al., 2007). In addition, FVT provides other indicators, theoretical maximal force/torque (F_0_/T_0_) and speed/cadence (v_0_) (Jaafar et al., 2016; Rudsits et al., 2018). Moreover, it allows to determine the optimal load (OPT_LOAD_) and optimal cadence (OPT_CAD_) of PPO generation (Dorel et al., 2005; Driss and Vandewalle, 2013; Rudsits et al., 2018). It is not surprising that several studies compared PPO obtained in the WAnT and FVT (Jaafar et al., 2016; Nikolaidis and Knechtle, 2021). Jaffar et al. (2016) compared the results of the WAnT (8.7% and 11% BM) and the F-v test and concluded that the optimal load during WAnT should be approximately equal to 10% BM for recreationally individuals. In the case of physically stronger people, FVT seems to be more appropriate in assessing mechanical peak power, and a load higher than 11% BM should be verified during the traditional WAnT (Jaafar et al., 2016). Interestingly, relatively less attention has been paid to the number of sprints used for FVT. For example, Kruger et al. (2020) used only two sprints for this purpose. In turn, Nikolaidis and Knechtle (2021) used four 7-s sprints with a load of 2, 3, 4, and 5 kg, MacIntosh et al. (2003) five 4–7-s sprints, while Rudsits et al. (2018) six 6-s sprints. In connection with this, justifies comparing the number of sprints used in the F-v and P-v models.

Therefore, the aims of the current investigation were twofold. Firstly, to examine estimated optimal load and PPO based on a FVT; secondly, to compare the PPO from the previous method with the Wingate Anaerobic Test (WAnT). The study used models based on a linear (F-v) and quadratic relationship (P-v and P-%BM), implementing a different number of sprints (three, four, five and nine). We assumed that the optimal load and peak power output determined in this approach would not differ between the three, four, five, and nine sprints used in the both models. Additionally, the PPO determined in the FVT will be higher than that measured during WAnT. Also, we hypothesized that the power derived from all P-%BM models is in agreement with the mechanical power output in the traditional WAnT.

## Materials and methods

### Participants

Participants were 15 academic level male athletes of different sports: football, basketball, handball, and racket sports (age 22.4 ± 2.3 years, body height 178.9 ± 6.8 cm; body weight 77.9 ± 12.2 kg; BMI 24.2 ± 2.4; %FAT 12.2% ± 3.5% and declared weekly physical activity was 6.0 ± 2.0 h). Each participant had at least 4 years of experience in training their discipline. All participants provided written informed consent to participate in the study. They were requested to wear comfortable exercise clothing, to avoid any heavy exercise, and to abstain from alcohol and caffeine 24 h before and between sessions. All testing sessions were carried out in the University Exercise Testing Laboratory (certificate PN–EN ISO 9001:2009). The experiment received a positive opinion from the Research Ethics Committee at the Wroclaw University of Health and Sport Sciences (6/2015).

### Study design

The subjects appeared in the laboratory four times, 72 h apart. The first visit included measuring anthropometric parameters and body composition by ultrasound. The 30-s Wingate Anaerobic Test (WAnT) was performed on a cycle ergometer. During the second, third, and fourth visits, a force-velocity test (FVT) was performed based on maximal 10-s sprints with a load of 3–11 kg in randomized order (summary participants performed nine all-out bouts). This study was carried out at a university testing laboratory for four sessions between 08:00–12:00 h, separated by 72 h. To minimize diurnal effects, participants were requested to visit at similar times.

### Anthropometric indicators

Body height and weight (BM) were measured on a WPT 200 medical scale (RADWAG, Radom, Poland). The BMI–Quetelet’s index (body weight∙body height-2) was calculated. Body composition was assessed using the BodyMetrix™ BX-2000 (IntelaMetrix, Livermore, United States) based on 2D ultrasound technology, using the Jackson and Pollock three-point scale. The measurement included the following points: on the chest, waist and front of the thigh. The computer program Body View Professional Software (IntelaMetrix, Livermore, United States) indicated the exact measurement locations. Body fat in the total body weight (%FAT) was automatically calculated after recorded subcutaneous adipose tissue thickness at each measurement site. After that, lean body mass (LBM) was calculated as the difference between body weight (BM) and fat mass, thus contains sum of muscles, bone and visceral mass. The device has been validated and provides a reliable measurement of the measured of body fat (Wagner and Teramoto, 2020).

### Wingate Anaerobic Test (WAnT)

The Wingate Anaerobic test was performed on the Ergomedic E894 cycle ergometer (Monark, Sweden). The test was preceded by a warm-up, which was carried out according to the recommendations of the creators of the test (Bar-Or, 1987). After the warm-up, the subjects remained in a sitting position on the cycle ergometer for 5 minutes. The flywheel load was 7.5% of the subject’s body weight. The effort lasted 30 seconds, and the subject’s task was to perform work with the maximal (possible) rotation frequency to achieve maximal power as quickly as possible and maintain it as long as possible. The test started from a standing position due to the possibility of obtaining higher power (MacIntosh et al., 2003). A standardized verbal motivation was provided to encourage maximal effort in all testing sessions. After the test, the subject remained on the cycle ergometer for 5 minutes for safety reasons. The cycle ergometer was controlled by a computer and MCE v.2.3 software (MCE, Poland), which calculated the total work (Wtot_WAnT_), peak power output (PPO_WAnT_), peak power output per kilogram of body weight (rPPO_WAnT_), time to obtain peak power output (tPPO_WAnT_) and fatigue index (FI_WAnT_). The highest cadence at the moment of generating maximal power was also calculated using the equation peakCAD_WAnT_ = PPO_WAnT_ ∙ load-1 (rpm), where the load is an individual load of 7.5% of body weight expressed in kilograms.

### Force-velocity relationship test (FVT)

This test was carried out on the cycle ergometer Ergomedic E894 (Monark, Sweden) and the warm-up was the same protocol as during the WAnT. The participants performed total nine sprints (three sessions with three sprints) with randomly selected load (3–11 kg) using Research Randomizer v4 (Urbaniak and Plous, 2013). The duration of each all-out sprint was 10-s, interspersed by 10-min passive recovery periods. Recovery time was set based on previous research published by Hottenrott et al. (2021), where they did not report any significant differences in peak power in four WAnTs performed with 10-min rest. An absolute load was used, which was converted as a percentage of total body weight (%BM) and lean body mass (%LBM) in calculations. The torque for each load was calculated based on Gardner et al. (2007) (Eq. 1):

$$Torque\;\left(N∙m\right)=\frac{Power\;\left(W\right)}{\left(Cadence\;\left(RPM\right)∙\frac{2\pi }{60}\right)}$$ (1)

A linear relationship was used to assess the maximal extrapolated torque (T_0_) and the extrapolated maximal cadence (v_0_). T_0_ was determined as the intersection points of the axes, and v_0_ of the *x*-axis as a function of T-v (Dorel et al., 2005). In order to determine the peak power output (PPO) in relation to cadence (model P-v), a second-degree polynomial function was used, expressed as a symmetrical hyperbola (Rudsits et al., 2018). Similarly, in the case of the PPO to %BM relationship (model P-%BM), which was used to determine the optimal load for PPO production (OPT_LOAD_). Relative PPO for body weight and lean body mass were also calculated. Based on the nine sprints performed in the FVT, further calculations were conducted using established three (3, 7 and 11 kg), four (4, 6, 8 and 10 kg), five (3, 5, 7, 9 and 11 kg) and nine sprints (all loads) in mathematical models. Hyperbolic curves were prepared for above mentioned three (curve_3_), four (curve_4_), five (curve_5_) and nine (curve_9_) implemented all-out sprints, respectively.

### Statistical analyses

The sample size was established *a priori* using G*Power 3.1 software (3.1.9.2, Germany) (Faul et al., 2007), the expected effect size was set at (Cohen’s f) 0.80, the α level was set at 0.05, and the power (1-β) was set at 0.9 (Cohen, 1988). The 15 participants in the group were necessarily recruited.

IBM SPSS Statistics version 26 software package (IBM, Inc., Chicago, United States) was used to statistically processing data. For each variable, the arithmetic mean ( $\bar{x}$ ) and standard deviation (SD) were determined. The Shapiro-Wilk test was used to assess the normality of the distribution of the examined features, and the homogeneity of variance was assessed with Levene’s test. One-way ANOVA was used to compare the extrapolated parameters: T_0_, v_0_, OPT_CAD_, rPPO, OPT_LOAD_ (%BM and %LBM) derived from all curves. The extrapolated PPO from both mathematical models were compared using two-way ANOVA with effect analysis MODEL (P-v vs. P-%BM) and CURVE (curve_3_, curve_4_, curve_5_, and curve_9_). Also, one-way ANOVA was used to determine differences between PPO measured in the WAnT and estimated in four tested curves. When a significant F ratio was obtained, the Bonferroni *post hoc* test was performed. The effect size was calculated for significant differences as partial eta-square (η^2^) (small = 0.01, moderate = 0.13, large = 0.26). To display the concordance between the PPO measured in the WAnT and estimated for 7.5% BM in mathematical models, Bland–Altman plots were constructed. Limits of agreement (LoA) were used to compare individual differences between variables. Mean differences ±1.96 SD were provided for LoA lines. The *p* < 0.05 level was considered statistically significant.

## Results

Table 1 shows the results obtained during 10-s sprints with loads ranging from 3 to 11 (kg).

**TABLE 1:** Mean and standard deviation ( $\bar{x}\pm SD$ ) of parameters measured during 10-s sprints with a load ranging from 3 to 11 kg.

Load (kg)	%BM (%)	W_TOT_ (kJ)	%W_WAnT_ (%)	peakCAD (rpm)	PPO (W)	tPPO (s)
Load_3_	3.9 ± 0.7	4.7 ± 0.4	24.4 ± 3.3	179.6 ± 13.3	538.9 ± 40.0	4.2 ± 1.3
Load_4_	5.3 ± 0.9	6.0 ± 0.6	31.2 ± 5.0	170.3 ± 14.1	681.2 ± 56.4	4.1 ± 0.6
Load_5_	6.6 ± 1.1	6.9 ± 0.8	35.7 ± 3.4	159.7 ± 15.8	798.6 ± 78.9	4.1 ± 0.7
Load_6_	7.9 ± 1.3	7.8 ± 1.1	39.8 ± 3.4	149.3 ± 17.2	895.8 ± 102.9	4.3 ± 1.0
Load_7_	9.2 ± 1.6	8.2 ± 1.2	42.1 ± 3.4	135.8 ± 18.8	950.6 ± 131.8	4.5 ± 1.1
Load_8_	10.5 ± 1.8	8.7 ± 1.5	44.2 ± 3.4	126.9 ± 19.5	1,015.0 ± 156.0	4.4 ± 1.0
Load_9_	11.8 ± 2.0	8.8 ± 1.8	44.7 ± 3.2	114.8 ± 20.1	1,033.0 ± 181.0	4.6 ± 1.1
Load_10_	13.1 ± 2.2	9.2 ± 2.3	46.2 ± 7.0	106.2 ± 26.2	1,061.8 ± 261.5	4.1 ± 0.9
Load_11_	14.3 ± 2.4	8.8 ± 2.8	43.6 ± 8.5	91.7 ± 28.5	1,008.5 ± 313.6	4.3 ± 1.4

%BM, the percentage of body mass, W_TOT_, total work, %W_WAnT_–the percentage of total work during 10″regarding WAnT, peakCAD, peak cadence, PPO, peak power output, tPPO, time to peak power output.

One-way ANOVA revealed that the extrapolated parameters were not statistically significant different between curve_3_, curve_4_, curve_5_ and curve_9_ (Table 2): T_0_ (F_3,56_ = 0.019, *p* = 0.99, η^2^ = 0.001); v_0_ (F_3,56_ = 0.20, *p* = 0.90, η^2^ = 0.01); OPT_CAD_ (F_3,56_ = 0.62, *p* = 0.61, η^2^ = 0.03), rPPO converted to BM (F_3,56_ = 0.062, *p* = 0.98, η^2^ = 0.003); and LBM (F_3,56_ = 0.065, *p* = 0.98, η^2^ = 0.003).

**TABLE 2:** Mean and standard deviation ( $\bar{x}\pm SD$ ) of parameters determined based on the F-v and P-v curve, taking into account three, four, five, and nine sprints.

Model	T_0_ (N∙m)	v_o_ (rpm)	OPT_CAD_ (rpm)	PPO (W)	rPPO (W∙kg_BM_-1)	rPPO (W∙kg_LBM_-1)
Curve3	192.5 ± 41.2	212.9 ± 13.6	106.1 ± 12.7	1,128.3 ± 273.0	14.6 ± 2.9	16.6 ± 3.2
Curve4	193.4 ± 40.1	216.1 ± 13.1	111.3 ± 13.7	1,134.2 ± 285.4	14.7 ± 3.1	16.7 ± 3.5
Curve5	190.6 ± 37.6	214.2 ± 13.6	106.2 ± 11.1	1,111.0 ± 247.7	14.4 ± 2.6	16.3 ± 2.9
Curve9	190.7 ± 37.1	215.8 ± 12.4	108.5 ± 10.4	1,102.9 ± 242.5	14.3 ± 2.7	16.2 ± 2.9

T_0_—maximal extrapolated torque, v_0_—maximal extrapolated velocity (cadence), OPT_CAD_, optimal cadence to produce peak power output, PPO, peak power output, rPPO, relative peak power regarding body mass or lean body mass.

**FIGURE 1:**
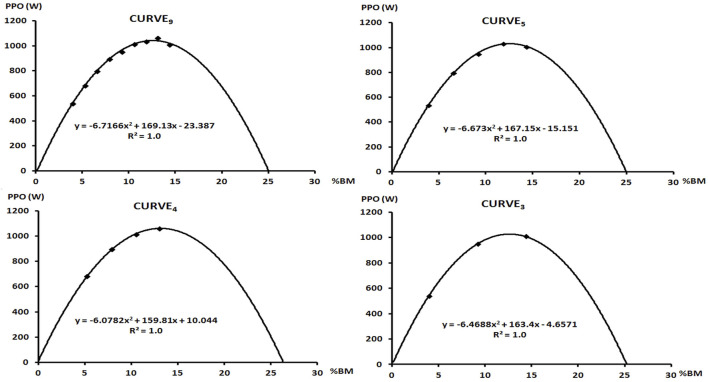
Curves showing the dependence of power to %BM depending on the number of sprints included in the model. The regression equation and coefficient of determination R^2^ were presented.

The PPO derived from model P-%BM was 1,114.4 ± 274.2 (W) for curve_3_, 1,138.7 ± 285.3 (W) for curve_4_, 1,105.2 ± 245.5 (W) for curve_5_ and 1,106.9 ± 244.6 (W) for curve_9_ (Figure 1). The two-way ANOVA revealed there was no main effect for MODEL (F_1,112_ = 0.003, *p* = 0.95, η^2^ = 0.000) and CURVE (F_3,112_ = 0.09, *p* = 0.96, η^2^ = 0.002). No MODEL × CURVE interaction was noted (F_3,112_ = 0.08, *p* = 0.99, η^2^ = 0.000). The optimal load related to percentage of body weight did not differ statistically significant (F_3,56_ = 0.174, *p* = 0.91, η^2^ = 0.01) and was 13.8 ± 3.2 (%BM), 14.1 ± 3.5 (%BM), 13.5 ± 2.8 (%BM) and 13.4 ± 2.6 (%BM) for curve_3_, curve_3_, curve_3_ and curve_9_, respectively. The optimal load expressed as a percentage of lean body mass was 15.8 ± 3.7 (%LBM), 16.0 ± 3.8 (%LBM), 15.4 ± 3.1 (%LBM), 15.2 ± 2.8 (%LBM) for three, four, five and nine sprints and did not significantly different (F_3,56_ = 0.172, *p* = 0.91, η^2^ = 0.009).

**FIGURE 2:**
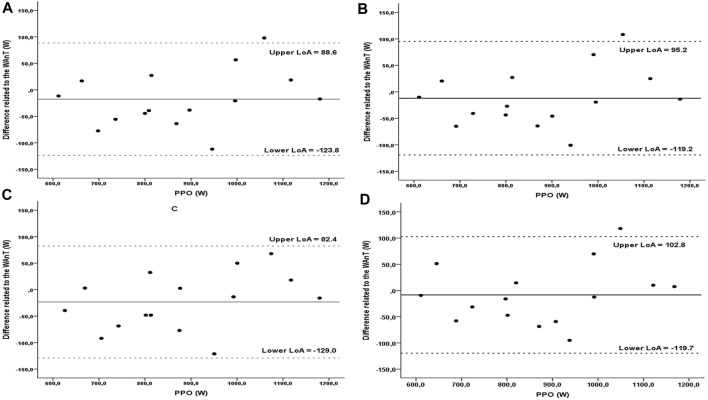
The Bland-Altman plot was used to define standard deviation, precision, and limits of agreement between the measurements of PPO measured in WAnT and determined with P-%BM. The measure differences (*y*-axis) are delineated as a two-measure mean function (*x*-axis) at PPO. The horizontal solid line represents the mean difference between the two measures (i.e., deviation). The two horizontal dotted lines represent the upper and the lower limit of agreement (1.96∙SD) of the mean difference between PPO in the WAnT and calculated in the curve_9_ **(A)**, curve_5_ **(B)**, curve_4_ **(C)** and curve_3_ **(D)**.

The subjects obtained the following results in WAnT: Wtot_WAnT_ = 19.8 ± 3.9 (kJ); PPO_WAnT_ = 870.6 ± 179.1 (W); rPPO_WAnT_ = 11.1 ± 1.0 (W∙kg_BM_^-1^); rPPO_WAnT_ = 12.8 ± 2.0 (W∙kg_LBM_^-1^); peakCAD_WAnT_ = 148.8 ± 23.9 (RPM); tPPO_WAnT_ = 4.6 ± 1.2 (s); FI_WAnT_ = 26.6 ± 5.9 (%). Two-way ANOVA revealed that PPO measured during WAnT was 27%–30% lower and differed significantly regardless of the number of sprints in the P-v model (F_4,70_ = 3.044, *p* = 0.02, η^2^ = 0.148). Similarly, for P-%BM (F_4,70_ = 2.976, *p* = 0.02, η^2^ = 0.145), where the PPO was 27%–31% higher compared to WAnT. To determine the power corresponding to 7.5% BM, the P-%BM model was used, and was 879.0 ± 164.8 (W), 893.9 ± 158.2 (W), 882.6 ± 162.6 (W), and 888.2 ± 162.6 (W), respectively for three, four, five and nine sprints implemented in the model. Moreover, extrapolated power for 7.5% BM did not differ significantly from those measured during the WAnT (F_4,70_ = 0.043, *p* = 0.99, η^2^ = 0.002) (Table 2). The coefficient of variation for curve_3_ was 3.7%, curve_4_ 3.9%, curve_5_ 3.7%, curve_9_ 3.8%. Bland-Altman analysis revealed a small bias for PPO_WAnT_ compared to the estimated power at 7.5% BM load found in curve_3_ (−8.4 W), curve_4_ (−23.3 W), curve_5_ (−12.0 W) and curve_9_ (−17.6 W) W) (Figure 2).

## Discussion

In this investigation we examined the estimated OPT_LOAD_ and PPO based on FVT, and compared peak power output from the previous method with the measured in the traditional WAnT (7.5% BM). The results of the present study found that the optimal load for producing peak power output was similar between the number of sprints and was greater than originally suggested. Additionally, in agreement with our hypothesis the PPO obtained during WAnT underestimated maximal anaerobic power by ∼30% compared with both examined models (P-v and P-%BM).

Finding the optimal load is crucial in the context of measuring the actual maximal power on the cycle ergometer (Linossier et al., 1996; Driss and Vandewalle, 2013). Underevaluating the load results in underestimating the power values ​​during WAnT by about 30% (870 W vs. 1100–1130 W). Finally, the power measured during this diagnostic test is not the real maximal power of the tested athlete but only the submaximal one, which is located on the arm of the F-v curve, not at its top. The results of our study indicate the OPT_LOAD_ of approx. 13.5%–14.0% of body weight for the diagnosis of true PPO, which is higher than in several other studies where 9%–11% of body weight were recommended (Jaafar et al., 2014; Vargas et al., 2015; Jaafar et al., 2016). We realize that we used the same load in kilograms for all the subjects, which constituted a different %BM or %LBM in individual cases. Similarly, Nikolaidis and Knechtle (2021) used the smaller load, i.e. 2, 3, 4, and 5 kg, in their study of football players. The constant %BM approach has been used by some researchers (Vargas et al., 2015; Krüger et al., 2020), and comparing the two methods could provide further insight into testing athletes’ maximal power. Reference should also be made to earlier research by Üçok et al. (2005), who suggested a 10%–11% load expressed in terms of LBM. The results of our study do not confirm these suggestions, indicating much higher 15%–16% LBM, depending on the used model. The level of anaerobic performance of the examined individuals may express these discrepancies. The OPT_LOAD_ generation of PPO is essential not only in diagnosing athletes (Linossier et al., 1996). It will be useful when performing repeated sprint training or sprint interval training, in which all-out efforts ≤10 s are used (Danek et al., 2020). Choosing the fitted load in such sessions can lead to more significant long-term adaptations.

In the current study, we determined the maximal power based on two different models, i.e., P-v and P-%BM, including three, four, five, and nine sprints in the calculations. The results clearly show that regardless of the PPO prediction method and the number of sprints, the peak power output does not differ. This proves the universality of the used procedures and, at the same time, indicates the time efficiency of the model based on three sprints, which is based on two extreme loads (3 and 11 kg) and one intermediate load (7 kg). According to Dunst et al. (2022), when creating correct maximal F-v profiles, obtaining different muscle recruitment patterns with points over a wide range of cadences is vital. At the same time, they indicated the need to use high fatigue-free cadences in the F-v models, which allow for a more accurate estimation of the maximal and optimal cadences (Dunst et al., 2022). This approach performed in a group of track cyclists considered cadences ranging from <50 to >200 (rpm). In the case of our tests, the extreme loads allowed us to achieve an average cadence in the range of 92–180 (rpm). Considering the application of the collected data for analysis, our research suggests that using more sprints in FVT does not bring additional benefits in modeling the F-v, P-v, and P-%BM profiles.

Several studies compared the peak power achieved during WAnT with that extrapolated from FVT (Jaafar et al., 2016; Nikolaidis and Knechtle, 2021). The PPO obtained in our study is higher than that determined based on FVT in the recreational group (884 ± 140 W), but lower than in the sports group (1,229 ± 136) (Jaafar et al., 2016). The results of maximal power similar to ours were presented by Nikolaidis and Knechtle (2021), comparing football players based on FVT (1,129 ± 222 W) and WAnT with a load of 7.5 body mass (846.8 ± 101.9 W). On the other hand, lower maximal power from FVT (949.25 ± 226.2 W) was reported by Vargas et al. (2015). That study was conducted in a group of recreationally physically active people, but the sprints were performed in a sitting position. Standing sprints allow for generating approx. 8%–12% higher PPO, which is associated with better power transfer from the upper body through the hips (Reiser et al., 2002). It is known that peak power output is produced at optimal cadence/speed and braking force (Vandewalle et al., 1987). The optimal cadence, calculated on the basis of our research, is in the range of 106–111 (rpm). At the same time, it is much lower than that obtained by the subjects during WAnT (∼149 rpm). The high cadence achieved during WAnT does not allow the generation of optimal torque (Forbes et al., 2014), which results in underestimated PPO (Driss and Vandewalle, 2013) and is reflected in our research. Jaafar et al. (2016) suggested slightly higher optimal cadences with FVT, ∼114 (rpm) in the recreational group and ∼119 (rpm) in the athlete group. The optimal speed (cadence) to generate peak power depends on the composition of the muscle fibers types (Sargeant et al., 1984). Higher optimal cadences have been associated with a higher proportion of fast twitch muscle fibers (Hautier et al., 1996; Linossier et al., 1996) and adenylate kinase activity (Linossier et al., 1996), which may explain the differences between these studies.

Regarding the last tested hypothesis, the extrapolated power at a load of 7.5% BM was compared to that measured during the 30-s WAnT, and any statistically significant differences were found. Each of the four models has a high coefficient of determination R^2^ >0.99, determined based on the regression equation, which indicates its mathematical correctness and high validity. Also, the Bland-Altman analysis showed agreement in each of the four tested models with the power measured during WAnT. The coefficient of variation (CV) of <4% in all the models demonstrates an acceptable level of variability in the results and is in line with the suggestions of other authors for power testing on a cycle ergometer (Van Praagh et al., 1992). Thus, the presented models can be used to determine the power with a certain %BM, e.g., in order to compare with the results of tests in which a different load was tested. Taking into account the time efficiency, the model consisting of three sprints is the most advantageous and can be carried out in one session.

It has been assumed that the WAnT is probably the most widely used protocol for evaluating maximal power due to its simplicity and short duration. FVT is time-consuming as it requires a rest period between sprints. If mechanical peak power output is the only parameter that is the target of the measurement and that has not been previously evaluated, FVT should be preferred (Jaafar et al., 2016). However, this does not allow the measurement of anaerobic performance and fatigue index, which on the other hand, indicates a possible advantage of WAnT over FVT (Jaafar et al., 2016). In this work, we did not determine the optimal load to generate the highest average power over the duration of WAnT, as in the case of Jaafar et al. (2016), but further research could consider these analyses. Furthermore, we agree with the reports of Jaafar et al. (2016), who suggested testing possible differences between optimal maximal and average power loads using loads higher than 11% BM in powerful participants. Although, we studied academic athletes who had not trained in cycling competitions, due to the universality of FVT, we decided also to present other key parameters (T_0_, v_0_, and OPT_CAD_). The results of our study are consistent with those previously published by Nikolaidis and Knechtle (2021) on football players. More commonly, however, these tests are used in diagnosing cyclists (Kordi et al., 2017; Dunst et al., 2022). First, the extrapolated T_0_ and v_0_ can be used to verify the optimal cadence and optimal torque, which are about 50% of the maximal values ​​(Douglas et al., 2021). Secondly, T_0_ (but not v_0_) correlates very strongly with PPO, which indicates the importance of maximal torque measurements as a factor strongly determining peak power (Kordi et al., 2017). Therefore, the test presented by us can be successfully used by cyclists.

### Practical applications

Using a load ∼13.5% BM among academic athletes allows for generating the highest PPO while reducing errors due to low external load and sub-optimal torque during testing. At the same time, use of models based on the relationships F-v, P-v, and P-%BM can be used to assess the maximal anaerobic performance, which may be particularly important in athletes practicing typical strength-speed disciplines whose efforts are based on the phosphagen energy system. During FVT, we used 10-s sprints, but considering the average PPO time of around 4–4.5 s in each of the used loads, this time could be reduced to ∼6 s. Different approaches can be found in the available literature, dominated by sprints lasting 4–15 s (MacIntosh et al., 2003; Vargas et al., 2015; Jaafar et al., 2016; Nikolaidis and Knechtle, 2021). If efforts longer than 10-s are used, PPO may be negatively affected as the subject may use a stimulation strategy (Gardner et al., 2007; Driss and Vandewalle, 2013). In this context, it is imperative to avoid fatigue in subsequent sprints during FVT. From a practical point of view, in the warm-up before WAnT, you can use three 6-s sprints with individual loads of 4, 9, and 14% of the body weight of the tested people, which results from the curve_3_ model, and then determine OPT_LOAD_, PPO, and other values. However, this approach should be tested in experimental studies. Moreover, Jaafar et al. (2016) prove that PPO from a single test (WAnT) did not greater than the power calculated using FVT for the same load. For this reason, we did not perform an additional test to verify the obtained PPO with an optimal load. However, such analyses will be the subject of further research.

### Limitations

This study has some limitations. It was carried out only on a group of 15 physically active men who were academic athletes. Further research are need to establish whether the F-v, P-v, and P-%BM models we propose could find application in different populations [i.e., children, women, sedentary and high-class athletes (cyclists), and the elderly] to check their universality. In addition, taking into account the use of only Monark ergometers in the tests, the models used by us can only be used on cycle ergometers of this company. The results obtained on other types cycle ergometers remain to be verified.

## Conclusion

Our results suggest the optimal load of ∼13.5% BW or ∼15.0% LBM when performing WAnT among academic male athletes. In the assessment of PPO based on the F-v, P-v, and P-%BM relationships, three sprints lasting a few seconds with a load of three, seven, and 11 kg can be implemented. This approach enables the determination of more important parameters, i.e., maximal torque, maximal and optimal cadence, and optimal load expressed as a percentage of total or lean body mass. In addition, based on these models, it is possible to determine the power of (any) load (relative to body weight) during traditional WAnT.

## Data Availability

The raw data supporting the conclusion of this article will be made available by the authors, without undue reservation.
